# Regional Minor Surgery

**Published:** 1903-09

**Authors:** 


Regional Minor Surgery.—Describing the Treatment of Those Conditions Daily Encountered by the General Practitioner. By George Gray Van Schoick, M. D., Attending Physician to the French Hospital, New York. Pages, 218. Price, $1.50. Published by the International Journal of Surgery Co., Medical Publishers, 100 William Street, New York. 1902.

This little volume impresses the reviewer that it was written by a practitioner and not by a theorist. Minor surgery is the everyday surgery of the average medical man, and because it is of everyday occurrence, the average surgeon becomes careless in his methods, and should read up occasionally a thoroughly active work on the subject, such as the one under consideration. The author has the faculty of saying what ought to be said, in a small space, hence the small number of pages in the book. As a small hand-book, there is nothing on the subject any better. T. J. B.

